# Inhibitory effects against α-glucosidase and α-amylase of the flavonoids-rich extract from *Scutellaria baicalensis* shoots and interpretation of structure–activity relationship of its eight flavonoids by a refined assign-score method

**DOI:** 10.1186/s13065-018-0445-y

**Published:** 2018-07-12

**Authors:** Ke Li, Fan Yao, Qiang Xue, Hang Fan, Lingguang Yang, Xiang Li, Liwei Sun, Yujun Liu

**Affiliations:** National Engineering Laboratory for Tree Breeding, College of Biological Sciences and Biotechnology, Beijing Forestry University, Qinghuadonglu No. 35, Haidian District, Beijing, 100083 China

**Keywords:** *Scutellaria baicalensis* shoots, Flavonoids, α-Glucosidase, α-Amylase, Structure–activity relationship, Refined assign-score method

## Abstract

A flavonoids-rich extract of *Scutellaria baicalensis* shoots and its eight high content flavonoids were investigated for their inhibitory effects against α-glucosidase and α-amylase. Results show that abilities of the extract in inhibiting the two enzymes were obviously higher than those of acarbose. Moreover, inhibitory abilities of all the eight individual flavonoids against the two enzymes show exactly a same order (i.e., apigenin > baicalein > scutellarin > chrysin > apigenin-7-*O*-glucuronide > baicalin > chrysin-7-*O*-glucuronide > isocarthamidin-7-*O*-glucuronide), and their structure–activity relationship could be well-interpretated by the refined assign-score method. Furthermore, based on the inhibitory abilities and their contents in the extract, it was found that the eight flavonoids made predominant contributions, among which baicalein and scutellarin played roles as preliminary contributors, to overall inhibitory effects of the extract against the two enzymes. Beyond these, contributions of the eight flavonoids to the overall enzyme inhibitory activity were compared with those to the overall antioxidant activity characterized in our recent study, and it could be inferred that within the basic flavonoid structure the hydroxyl on C-4′ of ring B was more effective than that on C-6 of ring A in enzyme inhibitory activities while they behaved inversely in antioxidant activities; scutellarin and apigenin contributed more to the overall enzyme inhibitory activity, and baicalin and scutellarin, to the overall antioxidant activity of the extract; and flavonoids of the extract, apart from directly inhibiting enzymes, might also be conducive to curing type 2 diabetes via scavenging various free radicals caused by increased oxidative stresses. 
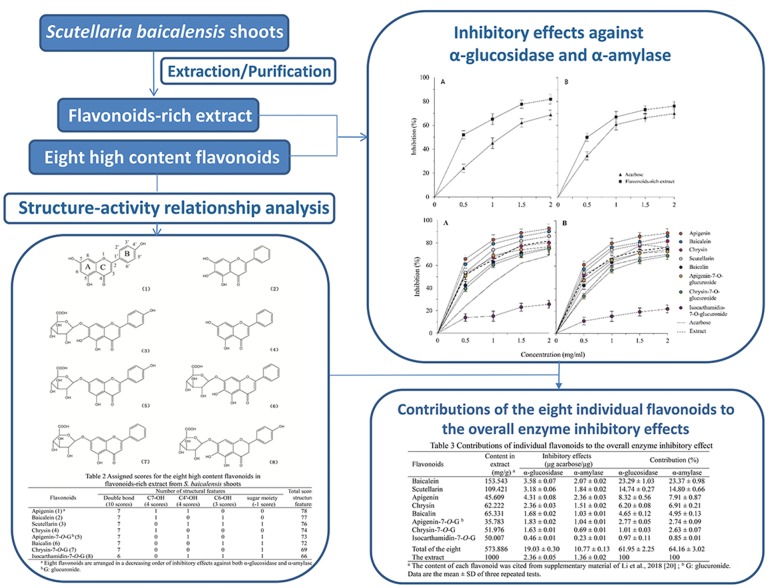

## Introduction

Diabetes is a chronic disease caused by deficiency in and insensitivity to insulin [1], usually resulting in postprandial hyperglycemia and various diabetic complications [2]. In 2013, 382 million individuals worldwide are living with diabetes, 90% of them were affected by non-insulin-dependent (type 2) diabetes, and the number is expected to rise to 592 million by 2035 [3, 4]. Diabetes has become a major cause of death in people younger than 60 years, and death caused by diabetes accounts for nearly 9% of the total global deaths [5]. Thus, it is urgent to explore effective therapeutic methods for diabetes and diabetic complications.

A promising approach for management of diabetes, particularly type 2 diabetes, is to decrease postprandial hyperglycemia by inhibiting carbohydrate hydrolyzing enzymes in gastrointestinal tract [6]. α-Amylase is involved in degrading long chain of starch and α-glucosidase breaks down oligosaccharides and disaccharides [7]. Inhibitors of these enzymes slow down carbohydrate digestion thus prolong overall digestion time, causing a reduction in glucose absorption and consequently blunting postprandial plasma glucose [8].

Currently there are several antidiabetic drugs such as acarbose that act by inhibiting α-amylase and α-glucosidase. Acarbose is an oligosaccharide of microbial origin (*Actinoplanes*) that potently inhibits in vitro and in vivo such brush-border enzymes as glucoamylase, dextrinase, maltase and sucrase as well as the pancreatic α-amylase [9]. Due to the presence of an intramolecular nitrogen, acarbose attaches to the carbohydrate binding site of α-glucosidase enzyme with an affinity exceeding that of the normal substrate by a factor of 10^4^–10^5^. The enzymatic reaction stops because the C–N linkage in the acarviosine unit of acarbose cannot be cleaved [10]. While efficient in attenuating the rise in blood glucose, continuous uses of acarbose and other similar drugs are often associated with undesirable effects [11]. It is for this reason that there is a need for natural α-glucosidase and α-amylase inhibitors that would possess no adverse or unwanted side effects. Traditional medicines have long employed herbal extracts as inhibitory agents against α-glucosidase and α-amylase [12] that, typically rich in polyphenolics, may own the potential in controlling postprandial hyperglycemia via their high antioxidant and/or enzymatic inhibitory effects [13, 14].

Flavonoids are a peculiar group of polyphenols ubiquitously distributed in plant kingdom and important functional compositions of human diets. Daily intake of flavonoids ranges between 50 and 800 mg/capita, depending mainly on consumptions of vegetables and/or fruits [15, 16]. Studies have suggested that flavonoids exhibit conspicuous biological activities [17–19], and attempts have been made in establishing a structure–activity relationship for a single type of effects such as antioxidant activities by the assign-score method [20]. Similar approaches should also be accordingly conducted on other biological activities of flavonoids, such as their hydrolytic enzyme inhibitory effects against α-amylase and α-glucosidase, in that establishment of structure–activity relationships of many such individual types of effects must be helpful to fully clarify the comprehensive structure–activity relationship of flavonoids. And this will certainly have some reference significance for establishing structure–activity relationship of other groups of bioactive compounds.

*Scutellaria baicalensis* in the family Labiatae, a perennial herb long listed in the Chinese Pharmacopoeia under the name “Huang Qin” in Chinese, is well-known for its root as medicine in East Asian countries [21]. Recently, pharmacological studies found that *S. baicalensis* shoot could also deliver a wide variety of beneficial therapeutic effects, such as cardiovascular protection, hepatoprotection, neuroprotection, anti-bacterial activity, improvement of memory deficits, and anti-tumor activity [22–24], indicating that it might be at least a good candidate of potential supplement for developing functional foods. Our previous study [20] identified fifteen flavonoids from the shoot of *S. baicalensis*, and eight high content flavonoids, including baicalin, baicalein, scutellarin, apigenin, chrysin, apigenin-7-*O*-glucuronide, chrysin-7-*O*-glucuronide, and isocarthamidin-7-*O*-glucuronide, were determined as main contributors to its antioxidant activities. Nevertheless, there are still no reports on anti-diabetic activities of the *S. baicalensis* shoot, let alone the contributions of individual compounds to these activities.

The objective of this study was to evaluate potentials of the flavonoids-rich extract, especially the contribution of the eight high content flavonoids, from *S. baicalensis* shoot as inhibitors against α-glucosidase and α-amylase, and to establish a structure–activity relationship for the eight flavonoids using the assign-score method, so as to providing base-line data of this valuable natural source for development of functional foods.

## Materials and methods

### Chemicals

Eight authentic standards (i.e., baicalin, baicalein, scutellarin, apigenin, chrysin, apigenin-7-*O*-glucuronide, chrysin-7-*O*-glucuronide, and isocarthamidin-7-*O*-glucuronide) were purchased from Institute for Control of Pharmaceutical and Biological Products (Beijing, China), acarbose, yeast α-glucosidase from *Saccharomyces cerevisiae*, porcine pancreatic α-amylase, and *p*-nitrophenyl-α-glucopyranoside (pNPG) were from Sigma-Aldrich Co. (St. Louis, MO, USA), and they were all stored at − 20 °C before using. All solvents (analytical grade) were bought from Beijing Chemical Factory, and purified water was from a mili-Q system (Millipore, Billerica, MA).

### Plant materials and extraction of flavonoids

Shoots of annual *S. baicalensis* were collected in Great Khingan, Heilongjiang, China, washed with purified water, air-dried till equilibrium humidity, and ground and stored at − 20 °C until extraction that was conducted as reported in [20]. Briefly, 250 g powder was refluxed for 2 h at 80 °C with purified water (plant materials: water = 1:10; w:v). The mixture was filtered through a Whatman No. 42 filter paper to obtain filtrate and the residues were subject to extraction twice more under the same conditions. All the filtrates (approximately 7500 mL) were combined and then evaporated under vacuum at 80 °C to obtain 500 mL brown concentrated extract solution. The extract solution, after adjusting to pH 3.1, was added onto a chromatographic column (45 mm × 450 mm), which was packed with 100 g AB-8 resins pretreated and activated according to the manufacturer’s recommendation. After getting adsorption equilibrium, the extract was desorbed with 1500 mL of 95% ethanol at a flow rate of 2 mL/min. Next, the eluent was evaporated under vacuum to dryness, and the extract, being characterized to be rich in flavonoids in our previous report [20], was collected and stored at − 20 °C for further analyses.

### Determinations of α-amylase inhibitory effect

α-Amylase inhibition activities of the flavonoids-rich extract and the eight authentic flavonoids demonstrated to be high content in the extract were determined as described by Liu et al. [25] with slight modifications. Briefly, 40 μL α-amylase (5 unit/mL) was mixed with 0.36 mL sodium phosphate buffer (0.02 M, pH 6.9 with 6 mM NaCl) and 0.2 mL sample (extract or each of the eight flavonoids) or acarbose (0, 0.5, 1.0, 1.5 and 2.0 mg/mL). After incubation for 20 min at 37 °C, 300 μL starch solution (1%) in sodium phosphate buffer (0.02 M, pH 6.9 with 6 mM NaCl) was added, and the mixture was re-incubated for 20 min, followed by addition of 0.2 mL dinitrosalicylic acid. The new mixture was then boiled for 5 min and cooled to room temperature. Cooled mixture was diluted by adding 10 mL distilled water, and absorbance was measured at 540 nm using a UV–visible spectrophotometer (Shimadzu UV-1700, Japan). Acarbose was used as a positive control, and inhibition of enzyme activity was calculated as follows: Inhibitory effect (%) = (OD_control_ − OD_sample_)/OD_control_ × 100. IC_50_ values were calculated by the logarithmic regression analysis.

### Determinations of α-glucosidase inhibitory effect

α-Glucosidase inhibitory effect was assayed as reported by Zhang et al. [26]. Briefly, 10 μL α-glucosidase (1 unit/mL) was mixed with 60 μL phosphate buffer (0.1 mM, pH 6.8) and 100 μL sample (extract or each of the eight flavonoids) or acarbose (0, 0.5, 1.0, 1.5, and 2.0 mg/mL) in corresponding well of a 96-well plate and the mixture was incubated for 10 min at 37 °C. Then, 30 μL pNPG solution (2 mM pNPG in 0.1 mM phosphate buffer) was added quickly to initiate the enzyme reaction. Absorbance was monitored at 405 nm every 15 min for 2 h using a microplate reader (Tecan infinite 200, Swiss). Inhibitory enzyme effect was determined by calculating the area under the curve (AUC) for each sample or acarbose and comparing the AUC with that of the negative control (0 mg/mL sample). Acarbose was used as a positive control and inhibition of enzyme activity was calculated as follows: Inhibitory effect (%) = (*A*_n_ − *A*_i_)/*A*_n_ × 100, where *A*_n_ is the AUC of negative control and *A*_i_ is the AUC of solution with inhibitors (sample or the positive control). In order to facilitate the subsequent analysis, the inhibitory effects of individual flavonoids and the flavonoids-rich extract were converted into acarbose equivalents, and the unit was accordingly expressed as ‘µg acarbose equivalents/µg’.

### The assign-score method refined for assessment of structure–activity relationship of flavonoids

Structure–activity relationship for eight individual flavonoids was performed using the assign-score method we established in a previous study [20], with slight refinements on specific scores assigned to different structural features of flavonoids. To be specific, we arbitrarily assigned different scores to the five structural features (see Fig. 3) reflecting their relatively importance to the inhibitory effects against α-glucosidase and α-amylase, i.e., double bonds (each 10 scores), hydroxyls on C-7 (each 4 scores), C-4′ (each 4 scores), and C-6 (each 3 scores), and sugar moieties (each-1 score). The minus mark indicates negative influence, indicating that the sugar moiety might be an attenuator to the enzyme inhibitory effect. A total score was calculated for each individual flavonoid, and a bigger score represents a higher inhibitory effect against the two enzymes studied.

### Statistical analyses

All experiments were conducted in triplicate, results were expressed as mean ± SD, and data were analyzed by SPSS software (version 17.0, Chicago, USA) and Excel 2016. Differences were considered to be significant at *p *< 0.05.

## Results and discussion

Flavonoids composition of the flavonoids-rich extract (total flavonoids content: 765.23 mg QE/g DW) from *S. baicalensis* shoots were investigated in our previous study [20]. Nineteen flavonoids were clearly detected with UPLC-Q-TOF–MS, and 15 were successfully identified. Quantitative determination by UPLC showed that the eight high content flavonoids accounted for 57.39% of the flavonoids-rich extract and 75.00% of its total flavonoids, and their order of contents from the highest to the lowest was: baicalein (153.543 mg/g) > baicalin (109.421) > scutellarin (65.331) > apigenin-7-*O*-glucuronide (62.222) > chrysin-7-*O*-glucuronide (51.976) > isocarthamidin-7-*O*-glucuronide (50.007) > apigenin (45.609) > chrysin (35.783). In addition, three of the eight flavonoids (baicalein, baicalin and scutellarin) were determined as primary active components of *S. baicalensis* shoots which made contributions of 58.33, 60.36 and 51.41% to overall antioxidant activities of the flavonoids-rich extract in DPPH, ABTS and CAA assays, respectively [20]. Thus, to explore the anti-diabetes activity of *S. baicalensis* shoots and the potential relationship of hypoglycemic effect and antioxidant activity, we analyzed the inhibitory effects against two key enzymes linked to type 2 diabetes (i.e., α-glucosidase and α-amylase) of the flavonoids-rich extract and its eight high content flavonoids in the present study.

### Inhibitory effects of the flavonoids-rich extract and its eight high content flavonoids against α-glucosidase and α-amylase

During the development of type 2 diabetes, insulin’s ability to stimulate cellular uptake of glucose from blood is compromised [27]. The most effective and beneficial therapy is to regain the optimal level of blood glucose as soon as possible after meal [28]. Thus inhibitors of both α-amylase that breaks down long-chain carbohydrates and α-glucosidase that catalyzes cleavage of glucose from disaccharide are effective in delaying glucose absorption and managing diabetes [29]. Acarbose is widely used in treatment of patients with type 2 diabetes via inhibiting the upper gastrointestinal glucosidases that convert complex polysaccharides into monosaccharides in a dose-dependent manner and result in a delayed glucose absorption and a depressed postprandial hyperglycemia. However, gastrointestinal side effects, mainly flatulence and sometimes soft stools or abdominal discomfort, have often been reported [30]. Inhibitory effects against α-amylase and α-glucosidase of the flavonoids-rich extract and its eight high content flavonoids were thus evaluated under these circumstances by taking acarbose as a positive control.

#### Inhibitory effects against α-glucosidase

**Fig. 1:**
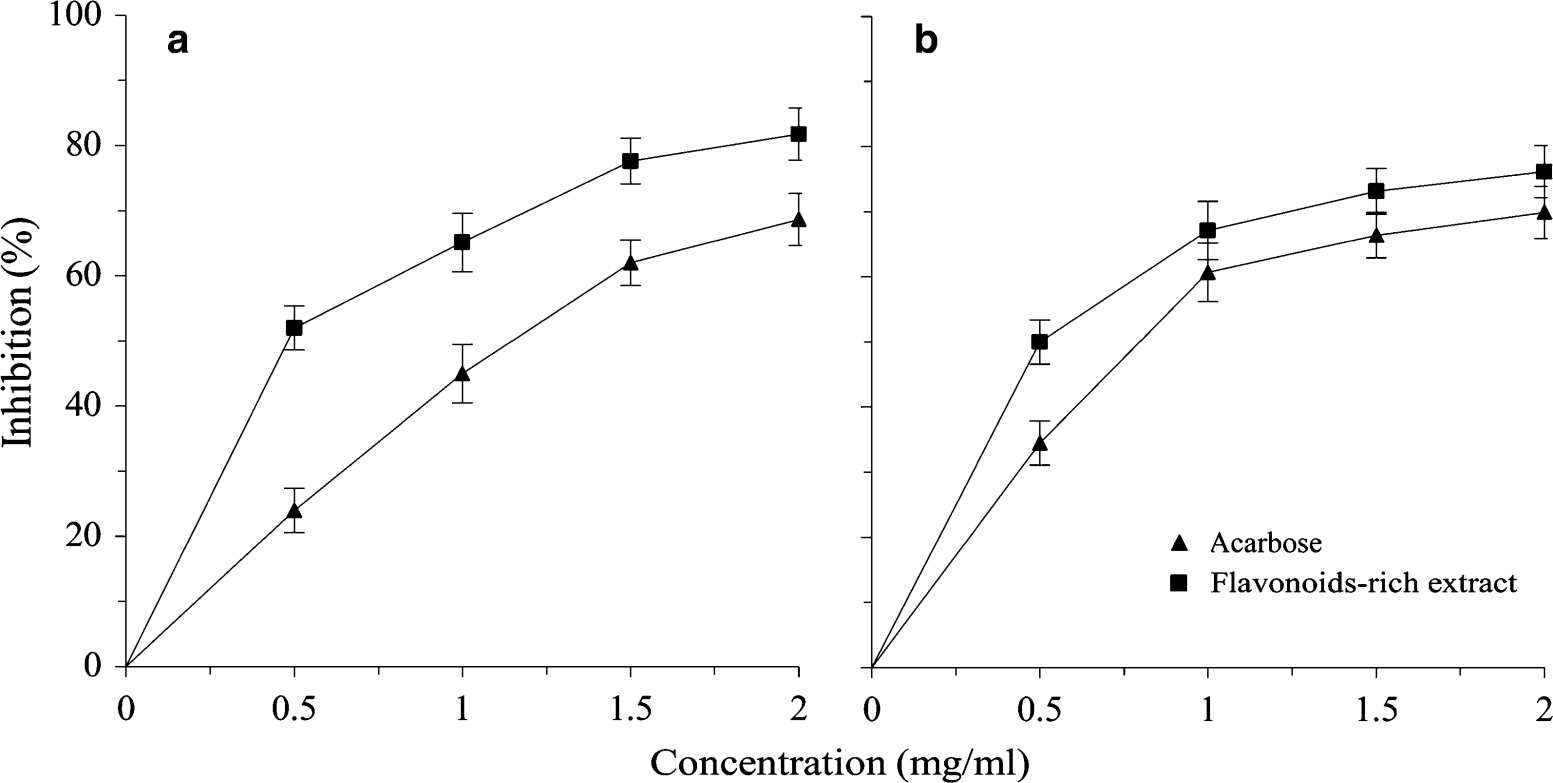
Inhibitory effects of the flavonoids-rich extract from *S. baicalensis* shoots at different concentrations (0, 0.5, 1, 1.5, and 2 mg/mL) against α-glucosidase (**a**) and α-amylase (**b**). Acarbose was used as the positive control to ensure that the results were reliable. Results were presented as mean ± SD of three independent experiments (n = 3)

As shown in Fig. 1a, effect of acarbose (the positive control), against α-glucosidase rose in a concentration-dependent manner, with an IC_50_ at 996.02 μg/mL (Table 1). At concentrations up to 1.5 mg/mL, the inhibitory effect increased near linearly, thereafter its increase slowed obviously, reaching a final inhibitory effect of 68.66% at 2 mg/mL. Effect of the flavonoids-rich extract, also rising in a concentration-dependent manner with an IC_50_ value at 421.54 μg/mL (Table 1), was significantly higher than that of acarbose at all concentrations (Fig. 1a). The effect initiated with a rapid increase up to 0.5 mg/mL, then became a relatively gradual increase from 0.5 to 1.5 mg/mL. The increase rate continued to decline thereafter, reaching a final inhibitory effect of 81.76% at 2 mg/mL. The results indicate that the flavonoids-rich extract from *S. baicalensis* shoots were much more effective in inhibiting the activity of α-glucosidase, therefore probably contained potentially potent compositions for treating the type 2 diabetes.

**Table 1:** IC_50_ values for enzyme inhibitory effects of the flavonoids-rich extract, eight flavonoids and acarbose

Flavonoids	IC_50_ (μg/mL)^a^	
	α-Glucosidase	α-Amylase
Acarbose	996.02 ± 21.34	678.43 ± 16.52
Extract	421.54 ± 10.01	498.59 ± 11.87
Apigenin	231.13 ± 5.35	287.53 ± 5.39
Baicalein	277.94 ± 6.21	336.22 ± 6.31
Scutellarin	313.25 ± 7.28	369.52 ± 8.43
Chrysin	422.67 ± 9.37	450.16 ± 10.45
Apigenin-7-*O*-G	543.28 ± 11.41	653.98 ± 15.28
Baicalin	591.58 ± 12.21	658.67 ± 16.38
Chrysin-7-*O*-G	612.13 ± 15.34	980.73 ± 18.34
Isocarthamidin-7-*O*-G	2149.78 ± 54.25	2941.25 ± 62.12

*G* glucuronide

^a^Data are the mean ± SD of three repeated tests

**Fig. 2:**
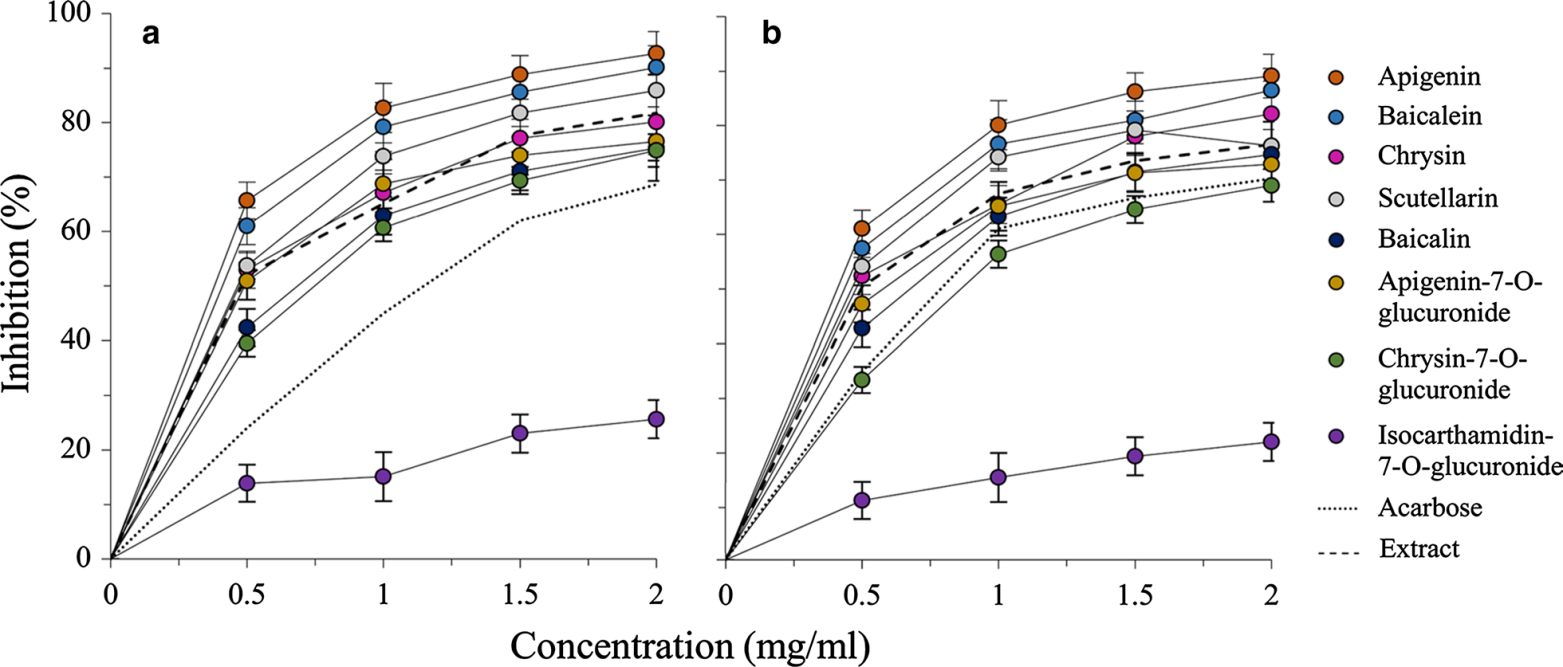
Inhibitory effects of the eight high content flavonoids found in the flavonoids-rich extract of *S. baicalensis* shoots at different concentrations (0, 0.5, 1, 1.5, and 2 mg/mL) against α-glucosidase (**a**) and α-amylase (**b**). Dashed and dotted curves represents those inhibitory effects of acarbose and the flavonoids-rich extract in respective reproduced from Fig. 1. Results are presented as mean ± SD of three independent experiments (n = 3)

For the eight high content flavonoids found in the extract (Fig. 2a), seven of them exhibited higher inhibitory effects against α-glucosidase than that of acarbose (see the dotted curve) at all concentrations with the highest inhibition at 92.7%, and they showed similar trends with that of the extract (see the dashed curve). Among the seven flavonoids, three (i.e., apigenin, baicalein and scutellarin) and two (i.e., baicalin and chrysin-7-*O*-glucuronide) exhibited respectively higher and lower effects than, and the other two (i.e., chrysin and apigenin-7-*O*-glucuronide) showed similar effects to, that of the extract. In contrast, dramatically different from these seven, the effect of isocarthamidin-7-*O*-glucuronide showed only weak and irregular increase from 0 to 2 mg/mL with the highest inhibition at only 25.64%. The same order of inhibitory effects of the eight flavonoids could also be reflected by the IC_50_ values show in Table 1. The results imply that these seven of the eight high content flavonoids, especially those three with even higher inhibitory effects than the extract, constituted the main composition in the extracts for inhibiting the α-glucosidase activity.

#### Inhibitory effects against α-amylase

From Fig. 1b, it is clear that effect of acarbose against α-amylase rose also in a concentration-dependent manner, with an IC_50_ at 678.43 μg/mL (Table 1). At concentrations up to 1 mg/mL, the inhibition increased quite abruptly, thereafter its increase slowed down, reaching an inhibition of 69.9% at 2 mg/mL. By comparison, effect of the extract showed a similar increase trend with but was apparently higher than that of acarbose at all concentrations, with the highest inhibition at 76.16% and an IC_50_ value at 498.59 μg/mL. The results also suggest that the flavonoids-rich extract was more effective than that of acarbose, especially at lower range of concentrations.

Inhibitory effects against α-amylase of the eight high content flavonoids showed roughly similar increasing patterns with those of acarbose and the extract (Fig. 2b). To be specific, one flavonoid (also isocarthamidin-7-*O*-glucuronide as shown in Fig. 2a) exhibited much lower, and another one (i.e., chrysin-7-*O*-glucuronide) only slightly lower than that of acarbose (Fig. 2b; see the dotted curve). The other six displayed higher effects than that of acarbose, among which four flavonoids, i.e., apigenin, baicalein, scutellarin and chrysin, exhibited even higher inhibitory effects than the extract (Fig. 2b; see the dashed curve). Furthermore, all these eight high content flavonoids presented the same inhibition order with that against α-glucosidase (Fig. 2a), which could also be reflected by the IC_50_ values (Table 1). The results also imply that the high content flavonoids, except isocarthamidin-7-*O*-glucuronide, especially those four with even higher inhibitory effects than that of the extract, consisted of the main composition in the extract for inhibiting the α-amylase activity.

It is worth noting that all samples, unlike the positive control acarbose, exhibited higher inhibitory effects against α-glucosidase than α-amylase (Table 1), being consistent with several previous reports [31–34]. Bischoff [9] has long reported that strong inhibition to α-glucosidase and mild inhibition to α-amylase of spice extracts could minimize the major setbacks of currently used α-glucosidase and α-amylase inhibitory drugs with side effects such as abdominal distention, flatulence, meteorism, and possibly diarrhea. Based on this argument, the flavonoids-rich extract from *S. baicalensis* shoots might also be effectively exploited in the management of postprandial hyperglycemia with minimal side effects.

### Structure–activity relationship of the eight high content flavonoids

Many flavonoids, such as rutin, myricetin, kaempferol and quercetin, have been previously reported to inhibit α-glucosidase and α-amylase, these flavonoids exhibit both hypoglycemic and antioxidant effects in diabetic animals [35–37], and their roles could be directly associated with their specific structural features, such as the position and number of hydroxyls and the number of double bonds on aromatic rings A and B as well as the heterocyclic ring C [38].

**Fig. 3:**
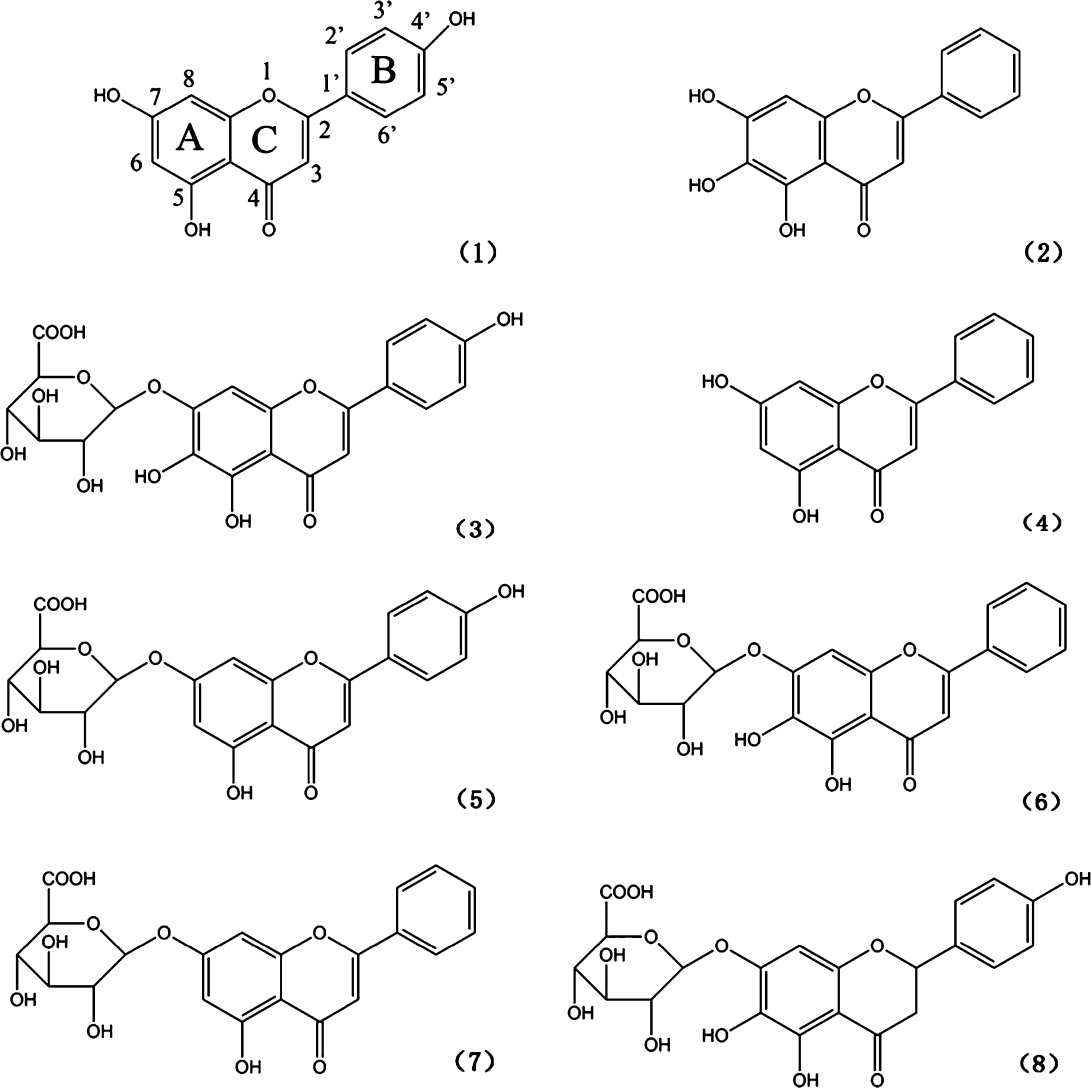
Chemical structures of the eight high content flavonoids arranged in a decreasing order of inhibitory effects against both α-glucosidase and α-amylase. (1) Apigenin; (2) baicalein; (3) scutellarin; (4) chrysin; (5) apigenin-7-*O*-glucuronide; (6) baicalin; (7) chrysin-7-*O*-glucuronide; (8) isocarthamidin-7-*O*-glucuronide

Figure 3 shows chemical structures of the eight high content flavonoids in a decreasing order of inhibitory effects against both α-glucosidase and α-amylase as revealed by data in Fig. 2 and Table 1. Structure–activity relationship for these flavonoids was then assessed using our refined assign-score method as described in the “Materials and methods” section.

As shown in Table 2 and Fig. 3, apigenin possessed the strongest enzyme inhibitory effect with a total score of 78, and this was attributed to seven double bonds in the two aromatic rings (7 × 10 = 70; a feature holding by all the high content flavonoids except the last one isocarthamidin-7-*O*-glucuronide) and hydroxyls existed on C-7 (4 scores) and C-4′ (4 scores). The relatively lower ability of baicalein with a total score of 77 was due to the existence of one hydroxyl on C-6 (3 scores) instead of C-4′ (4 scores). As to the third strongest scutellarin (76 scores), it possesses the same structure with the second strongest baicalein except for a sugar moiety at position C-7 (− 1 score), causing further decrease of its inhibitory effects against the two enzymes.

**Table 2:** Assigned scores for the eight high content flavonoids in flavonoids-rich extract from *S. baicalensis* shoots

Flavonoids	Number of structural features					Total score of structural features
	Double bond (10 scores)	C7-OH (4 scores)	C4′-OH (4 scores)	C6-OH (3 scores)	Sugar moiety (− 1 score)	
Apigenin (1)^a^	7	1	1	0	0	78
Baicalein (2)	7	1	0	1	0	77
Scutellarin (3)	7	0	1	1	1	76
Chrysin (4)	7	1	0	0	0	74
Apigenin-7-*O*-G (5)	7	0	1	0	1	73
Baicalin (6)	7	0	0	1	1	72
Chrysin-7-*O*-G (7)	7	0	0	0	1	69
Isocarthamidin-7-*O*-G (8)	6	0	1	1	1	66

*G* glucuronide

^a^Eight flavonoids are arranged in a decreasing order of inhibitory effects against both α-glucosidase and α-amylase

Chrysin (74 scores), apigenin-7-*O*-glucuronide (73 scores) and baicalein (72 scores) are all found to have two hydroxyls. Although baicalin (i.e., baicalein-7-*O*-G) and apigenin-7-*O*-glucuronide both carry a sugar moiety at C-7 (− 1 score), the hydroxyl on C-4′ of apigenin-7-*O*-glucuronide (4 scores) make it possessing higher inhibitory effect than that of baicalin, which carries a second hydroxyl on C-6 (3 scores) (Table 2 and Fig. 3).

Lastly, chrysin-7-*O*-glucuronide (69 scores) and isocarthamidin-7-*O*-glucuronide (66 scores) shows much weaker inhibitory effects. Although the number of hydroxyls presented in chrysin-7-*O*-glucuronide (1 hydroxyl) were less than that of isocarthamidin-7-*O*-glucuronide (3 hydroxyls), the former showed still higher inhibitory effect than that of the latter. This was attributed to lack of a double bond (10 scores) between C-2 and C-3 in the heterocyclic ring C of the latter (isocarthamidin-7-*O*-glucuronide) (Table 2 and Fig. 3).

Collectively, our findings that based on the refined assign-score method commendably demonstrated that α-glucosidase and α-amylase inhibitory effects of the eight flavonoids were highly tied to their structural features. Specifically, double bonds between C-2 and C-3 might be an essential factor, and hydroxyls on rings A (C-7 and C-6) and B (C-4′) are augmentors, and sugar moiety is an attenuator influencing enzyme inhibitory effect (Fig. 3). Interestingly, the antioxidant activities of the eight flavonoids demonstrated in our previous study [20] were also highly tied to these structural features, although their orders in the two activities (i.e., antioxidant activities and enzyme inhibitory effects) are not exactly the same: baicalein and baicalin showed higher antioxidant activities but lower enzyme inhibitory effects than those of apigenin and apigenin-7-*O*-glucoside, respectively. Obviously, changes of the above four flavonoids in the two orders might result from different positions of hydroxyls on rings A and B, thus it can be inferred that the hydroxyl on ring A (C-6) is more effective than that on ring B (C-4′) in antioxidant activities. On the contrary, the hydroxyl on ring B (C-4′) is more effective than that on ring A (C-6) in enzyme inhibitory effects (Table 2 and Fig. 3).

### Contributions of the eight individual flavonoids to the overall enzyme inhibitory effect

To determine contributions of the eight individual flavonoids to overall enzyme inhibitory effect of the flavonoids-rich extract from *S. baicalensis* shoots, a calculation formula was developed as follows: Contribution (%) = [E_i_/E_0_] × *C* × 100, where Ei and E_0_ are enzyme inhibitory effects of an individual flavonoid and the flavonoids-rich extract, respectively, on the base of acarbose equivalent, and *C* is the content of an individual flavonoid in the extract in mg/g. Table 3 shows that the eight high content flavonoids made strong contributions to the overall enzyme inhibitory activities of the flavonoids-rich extract against both the α-glucosidase and α-amylase (61.95 and 64.16%, respectively). And the two orders of contributions are exactly the same, i.e., baicalein > scutellarin > apigenin > chrysin > baicalin > apigenin-7-*O*-glucuronide > chrysin-7-*O*-glucuronide > isocarthamidin-7-*O*-glucuronide (Table 3). In particular, baicalein and scutellarin provided major contributions to those of the eight flavonoids (61.39 and 59.54%), which also accounted for 38.03 and 38.17% of the overall enzyme inhibitory effects, respectively.

**Table 3:** Contributions of individual flavonoids to the overall enzyme inhibitory effect

Flavonoids	Content in extract (mg/g)^a^	Inhibitory effects (μg acarbose/μg)		Contribution (%)	
		α-glucosidase	α-amylase	α-glucosidase	α-amylase
Baicalein	153.543	3.58 ± 0.07	2.07 ± 0.02	23.29 ± 1.03	23.37 ± 0.98
Scutellarin	109.421	3.18 ± 0.06	1.84 ± 0.02	14.74 ± 0.27	14.80 ± 0.66
Apigenin	45.609	4.31 ± 0.08	2.36 ± 0.03	8.32 ± 0.56	7.91 ± 0.87
Chrysin	62.222	2.36 ± 0.03	1.51 ± 0.02	6.20 ± 0.08	6.91 ± 0.21
Baicalin	65.331	1.68 ± 0.02	1.03 ± 0.01	4.65 ± 0.12	4.95 ± 0.13
Apigenin-7-*O*-G	35.783	1.83 ± 0.02	1.04 ± 0.01	2.77 ± 0.05	2.74 ± 0.09
Chrysin-7-*O*-G	51.976	1.63 ± 0.01	0.69 ± 0.01	1.01 ± 0.03	2.63 ± 0.07
Isocarthamidin-7-*O*-G	50.007	0.46 ± 0.01	0.23 ± 0.01	0.97 ± 0.11	0.85 ± 0.01
Total of the eight	573.886	19.03 ± 0.30	10.77 ± 0.13	61.95 ± 2.25	64.16 ± 3.02
The extract	1000	2.36 ± 0.05	1.36 ± 0.02	100	100

Data are the mean ± SD of three repeated tests

*G*: glucuronide

^a^ The content of each flavonoid was cited from supplementary material of Li et al. [20]

It is worth noting that, as contents of individual flavonoids in the extract were different, their order of contributions to the overall activity was quite different with that of individual inhibitory ability. For instance, apigenin, although it was only the third contributor due to its second lowest content (Table 3), was the most effective flavonoid in the inhibitory ability (Table 1). In contrast, baicalein, which displayed a lower inhibitory ability than apigenin (Table 1), was the biggest contributor to the overall inhibitory activity of the extract (Table 3). Furthermore, baicalein and scutellarin kept at high positions in all the three orders, namely, enzyme inhibitory ability (Table 1) and their contents in and contributions to the extract (Table 3), demonstrating that these two flavonoids could be regarded as the primary flavonoids in the flavonoids-rich extract from *S. baicalensis* shoots in the inhibitory effects against α-glucosidase and α-amylase.

By comparing the contributions to overall enzyme inhibitory effects of the flavonoids-rich extract demonstrated by the current study with those to overall antioxidant activities reported in our previous work [20], we found that the eight flavonoids provided higher contribution in antioxidant activity (75.85% in average of the three assays, i.e., DPPH, ABTS and CAA) than that in enzyme inhibitory effects (63.04% in average against the two enzymes). In view of the eight individual flavonoids in the two orders of contribution, the first (baicalein), the fourth (chrysin) and the last three (apigenin-7-*O*-glucuronide, chrysin-7-*O*-glucuronide and isocarthamidin-7-*O*-glucuronide) were the same, thus differences occurred only with the other three, namely, scutellarin > apigenin > baicalin in enzyme inhibitory, and baicalin > scutellarin > apigenin in antioxidant abilities, indicating that scutellarin and apigenin contributed more to the overall enzyme inhibitory ability, and baicalin and scutellarin, to the overall antioxidant ability of the extract.

These days, more and more attentions are focusing on natural products that may be benefit to the intractable type 2 diabetes. According to current opinions, it is believed that inhibitory effects against the two key enzymes, namely, α-amylase and α-glucosidase, can significantly decrease the postprandial increase of blood glucose level after a mixed carbohydrate diet [11, 39–42]. In the present study, the flavonoids-rich extract from *S. baicalensis* shoots showed high inhibitory effects against both α-glucosidase and α-amylase (Figs. 1, 2 and Table 1), revealing that it could implement potential anti-diabetes function by inhibiting the two enzymes. Furthermore, it has been reported that many natural food sources (such as vegetables and fruits) and traditional medicinal herbs that are rich in phenolic compounds, especially flavonoids, showed strong interaction with proteins and could inhibit their enzymatic activities by forming complexes and changing conformations [43]. Recent studies further demonstrated that small less-polar phenolic compounds including flavonoids could easily interact with hydrophobic amino acid residues near active sites of the targeted enzymes, which might strongly cause inhibitory effects against various glucosidases [44]. In our study, double bonds, hydroxyls on rings A (C-7 and C-6) and B (C-4′) and sugar moiety of the eight high content individual flavonoids were proved to be important factors in influencing enzyme inhibitory effect (Fig. 3 and Table 2), however, the specific reaction mechanisms with respect to influences on and interaction with active sites of the relevant enzymes still need to be further investigated.

In addition, increased oxidative stress is widely accepted as a participant in the development and progression of diabetes [45]. Abnormally high levels of free radicals and simultaneous decline of antioxidant defense mechanisms could lead to damage of cellular organelles and enzymes, increased lipid peroxidation, and development of insulin resistance [46]. Li et al. [47] also outlined that antioxidant effects of flavonoids increased cell membrane stability and protected them from damage, which participates in increasing insulin sensitivity and inhibits free radical generation. By comparing enzyme inhibitory effect with antioxidant activity, it is easy to figure out that orders in the two set criterions of the eight high content flavonoids in the flavonoids-rich extract described above were similar but not exactly the same, and more vigorous contributions of the eight flavonoids were found to the antioxidant capacities than to the enzyme inhibitory effects (Table 3). Following this line of thinking, it is not difficult to draw inferences as that flavonoids of the extract, apart from directly inhibiting glycosidases such as α-amylase and α-glucosidase, might also be conducive to curing the intractable type 2 diabetes via scavenging various free radicals resulted from increased oxidative stresses, which is also worthy of further elucidation.

## Conclusions

In the present study, flavonoids-rich extract from *S. baicalensis* shoots showed high α-glucosidase and α-amylase inhibitory effects with IC_50_ values at 421.54 and 498.59 μg/mL, respectively. The inhibitory ability order of its eight high content flavonoids against both α-glucosidase and α-amylase was apigenin > baicalein > scutellarin > chrysin > apigenin-7-*O*-glucuronide > baicalin > chrysin-7-*O*-glucuronide > isocarthamidin-7-*O*-glucuronide. The structure–activity relationship further revealed that double bonds between C-2 and C-3 on ring C might be essential effectors, and hydroxyls on rings A (C-7 and C-6) and B (C-4′) were augmentors, and sugar moiety was an attenuator influencing enzyme inhibitory capacity. In addition, we found that the eight flavonoids made contributions of 61.95 and 64.16% to overall activities in the two assays, respectively. Among the eight flavonoids, baicalein and scutellarein were not only the higher content components but the superior contributors. Accordingly, the eight high content flavonoids were the predominant contributors, and baicalein and scutellarein were defined as the primary contributors in the flavonoids-rich extract from *S. baicalensis* shoots. Furthermore, by comparing these results with those in our previous study [20], it was inferred that the hydroxyl on ring B (C-4′) is more effective than that on ring A (C-6) in enzyme inhibitory effects while the hydroxyl on ring A (C-6) is more effective than that on ring B (C-4′) in antioxidant activities; scutellarin and apigenin contributed more to the overall enzyme inhibitory ability, and baicalin and scutellarin, to the overall antioxidant ability of the extract; and flavonoids of the extract, apart from directly inhibiting glycosidases such as α-amylase and α-glucosidase, might also be conducive to curing type 2 diabetes via scavenging various free radicals resulted from increased oxidative stresses. Our findings provide useful information for further development of *S. baicalensis* shoots as potential supplements for various functional foods.
